# Supplementary material for: Preserving the spots: Jaguar (Panthera onca) distribution and priority conservation areas in Colombia
Source: PLoS One. 2024 Mar 22;19(3):e0300375. doi: 10.1371/journal.pone.0300375 (PMC10959345; doi:10.1371/journal.pone.0300375)
Supplement: S1 Table — (DOCX) [file pone.0300375.s001.docx]

**Preserving the spots: Jaguar (*Panthera onca*) distribution and priority conservation areas in Colombia**

María Camila Machado-Aguilera, Leonardo Lemus-Mejía, Jairo Pérez-Torres, Diego A. Zárrate-Charry, Andrés Arias-Alzate, José F. González-Maya.

**SUPPORTING INFORMATION**

**S1 TABLE**

**Supporting Information 1 (S1 Table).** Records used for species distribution modeling of *Panthera onca* in Colombia classified according to filters for credibility and geographic precision

| Source | Lon | Lat | Department | Municipality | Credibility category | Geographic category |
| --- | --- | --- | --- | --- | --- | --- |
| GBIF | -67.0667 | 1.9167 | Guainia | San Felipe | high | medium |
| GBIF | -72.3936 | 0.6166 | Caqueta | Solano | low | low |
| GBIF | -67.8576 | 5.2625 | Vichada | Cumaribo | low | medium |
| GBIF | -73.361486 | 3.229723 | Meta | Puerto Lleras | high | medium |
| GBIF | -67.849722 | 4.628056 | Vichada | Cumaribo | high | high |
| GBIF | -73.959772 | 4.597286 | Cundinamaca | Choachi | high | low |
| GBIF | -73.784507 | 2.893078 | Meta | Vistahermosa | high | low |
| GBIF | -70.039704 | -3.967681 | Amazonas | Leticia | high | low |
| GBIF | -73.784507 | 2.893078 | Meta | Vistahermosa | high | low |
| GBIF | -70.039704 | -3.967681 | Amazonas | Leticia | high | low |
| GBIF | -73.784507 | 2.893078 | Meta | Vistahermosa | high | low |
| GBIF | -76.91142 | 1.229191 | Putumayo | Sibundoy | high | high |
| GBIF | -76.91142 | 1.229191 | Putumayo | Sibundoy | high | high |
| GBIF | -73.784507 | 2.893078 | Meta | Vistahermosa | high | low |
| GBIF | -73.959772 | 4.597286 | Cundinamaca | Choachi | high | low |
| GBIF | -70.039704 | -3.967681 | Amazonas | Leticia | high | low |
| GBIF | -76.91142 | 1.229191 | Putumayo | Sibundoy | high | high |
| GBIF | -76.91142 | 1.229191 | Putumayo | Sibundoy | high | high |
| GBIF | -75.15 | 7.07 | Antioquia | Anori | medium | high |
| GBIF | -75.77 | 7.18 | Antioquia | Ituango | medium | high |
| GBIF | -75.88 | 5.66 | Antioquia | Andes | medium | medium |
| GBIF | -74.87 | 7.5 | Antioquia | Zaragoza | medium | high |
| GBIF | -73.833333 | 3.433333 | Meta | Granada | high | medium |
| GBIF | -72.996559 | 3.49714 | Meta | San Martin | high | medium |
| GBIF | -75.096634 | 0.530457 | Caqueta | Solano | high | low |
| GBIF | -73.309107 | 11.272506 | La Guajira | Dibulla | high | high |
| Literature_review | -76.0838889 | 8.00388889 | Cordoba | Tierralta | medium | high |
| Literature_review | -75.9666667 | 7.98444444 | Cordoba | Montelibano | medium | medium |
| Literature_review | -74.49925 | 6.67686111 | Antioquia | Puerto Berrio | medium | high |
| Literature_review | -74.88067 | 6.17363 | Antioquia | San Carlos | high | high |
| Literature_review | -72.28333 | -0.4 | Caqueta | Solano | high | low |
| Literature_review | -73.25 | 10.48333 | Cesar | Valledupar | high | low |
| Literature_review | -73.85 | 3.45 | Meta | Granada | high | low |
| Literature_review | -72.28333 | -0.4 | Caqueta | Solano | high | low |
| Literature_review | -73.85 | 3.45 | Meta | Granada | high | low |
| Literature_review | -73.25 | 10.48333 | Cesar | Valledupar | high | low |
| Literature_review | -71.73694 | -2.13333 | Amazonas | Puerto Arica | low | low |
| Literature_review | -72.11667 | -2.08861 | Amazonas | La Chorrera | low | low |
| Literature_review | -69.5 | -1.09333 | Vaupes | Taraira | low | low |
| Literature_review | -72.26667 | -0.61417 | Caqueta | Solano | low | low |
| Literature_review | -71.0025 | 0.01806 | Vaupes | Pacoa | low | low |
| Literature_review | -75.21667 | 0.45 | Putumayo | Puerto Leguizamo | low | low |
| Literature_review | -71.99889 | 0.65639 | Caqueta | Solano | low | low |
| Literature_review | -68.19 | 2.16667 | Guainia | Puerto Colombia | low | low |
| Literature_review | -68.19 | 2.16667 | Guainia | Puerto Colombia | low | low |
| Literature_review | -68.17 | 2.20167 | Guainia | Puerto Colombia | low | low |
| Literature_review | -77.34628 | 2.77017 | Cauca | Timbiqui | high | low |
| Literature_review | -73.88944 | 3.34639 | Meta | San Juan de Arama | low | low |
| Literature_review | -73 | 3.5 | Meta | San Martin | low | low |
| Literature_review | -68.36667 | 3.88333 | Guainia | Inirida | low | low |
| Literature_review | -75.88 | 5.66 | Antioquia | Andes | high | low |
| Literature_review | -75.15 | 7.07 | Antioquia | Anori | high | low |
| Literature_review | -75.77 | 7.18 | Antioquia | Ituango | high | low |
| Literature_review | -74.87 | 7.5 | Antioquia | Zaragoza | high | low |
| Literature_review | -67.84972 | 4.62806 | Vichada | Cumaribo | high | low |
| Literature_review | -73.83333 | 3.43333 | Meta | Granada | high | low |
| Literature_review | -73.83333 | 3.43333 | Meta | Granada | low | low |
| Literature_review | -75.1 | 0.53333 | Caqueta | Solano | high | low |
| Literature_review | -75.1 | 0.53333 | Caqueta | Solano | low | low |
| SIB | -71.6043361 | 6.1995 | Casanare | Hato Corozal | medium | high |
| Species Link | -72.267 | -0.614167 | Caqueta | Solano | medium | high |
| Species Link | -72.1167 | -2.08861 | Amazonas | La Chorrera | medium | high |
| Species Link | -68.367 | 3.883 | Guainia | Inirida | medium | high |
| Species Link | -73.88945 | 3.346389 | Meta | San Juan de Arama | medium | high |
| Species Link | -68.19 | 2.1667 | Guainia | Puerto Colombia | medium | high |
| Species Link | -71.73694 | -2.133 | Amazonas | Puerto Arica | medium | medium |
| Species Link | -68.17 | 2.20167 | Guainia | Puerto Colombia | medium | high |
| Species Link | -71.9989 | 0.656389 | Caqueta | Solano | medium | low |
| Species Link | -75.2167 | 0.45 | Putumayo | Puerto Leguizamo | medium | high |
| Species Link | -71.0025 | 0.018056 | Vaupes | Pacoa | medium | medium |
| Species Link | -68.19 | 2.1667 | Guainia | Puerto Colombia | medium | high |
| Species Link | -69.5 | -1.093 | Vaupes | Taraira | medium | high |
| Regional_mammalogist | -75.0194 | 7.0428 | Antioquia | Amalfi | medium | high |
| Regional_mammalogist | -71.970932 | 1.134471 | Guaviare | Miraflores | medium | high |
| Regional_mammalogist | -67.872934 | 3.843892 | Guainia | Inirida | medium | high |
| Regional_mammalogist | -72.478948 | 2.35371 | Guaviare | El Retorno | medium | medium |
| Regional_mammalogist | -73.825 | 1.7 | Meta | La Macarena | medium | high |
| Regional_mammalogist | -73.66432 | 2.827859 | Meta | Vistahermosa | medium | high |
| Regional_mammalogist | -73.393378 | 2.489181 | Meta | Vistahermosa | medium | high |
| Regional_mammalogist | -77.13824 | 2.63087 | Cauca | El Tambo | medium | high |
| Regional_mammalogist | -76.365609 | 5.114023 | Choco | Condoto | medium | high |
| Regional_mammalogist | -74.014505 | 6.371224 | Santander | Cimitarra | medium | high |
| Regional_mammalogist | -76.36944 | 6.517915 | Antioquia | Urrao | medium | high |
| Regional_mammalogist | -74.234 | 7.7632 | Bolivar | Santa Rosa del Sura | medium | medium |
| Regional_mammalogist | -70.853296 | -1.202404 | Amazonas | Miriti - Parana | medium | high |
| Regional_mammalogist | -75.072539 | 9.903745 | Bolivar | San Juan Nepomuceno | medium | high |
| Regional_mammalogist | -74.732298 | 9.944146 | Magdalena | Tenerife | medium | high |
| Regional_mammalogist | -73.462725 | 10.700916 | Cesar | Valledupar | medium | high |
| Regional_mammalogist | -72.816987 | 10.781378 | La Guajira | Fonseca | medium | high |
| Regional_mammalogist | -72.369699 | 11.263073 | La Guajira | Maicao | medium | high |
| Regional_mammalogist | -75.85301 | 4.702978 | Valle del Cauca | Cartago | medium | low |
| Regional_mammalogist | -76.76349 | 3.782281 | Valle del Cauca | Dagua | medium | low |
| Regional_mammalogist | -67.870075 | 4.520413 | Vichada | Cumaribo | medium | high |
| Regional_mammalogist | -76.63 | 7.585277 | Antioquia | Chigorodo | medium | high |
| Regional_mammalogist | -71.75 | 6.7 | Arauca | Tame | medium | high |
| Regional_mammalogist | -77.638611 | 2.281944 | Cauca | Guapi | medium | high |
| Regional_mammalogist | -78.840555 | 1.643055 | Nariño | San Andres de Tumaco | medium | high |
| Regional_mammalogist | -72.431388 | 8.188611 | Norte de Santander | San Jose de Cucuta | medium | high |
| Regional_mammalogist | -73.483333 | 6.266666 | Santander | Contratacion | medium | high |
| Regional_mammalogist | -75.233333 | 9.3 | Sucre | San Juan de Betulia | medium | high |
| Regional_mammalogist | -77.1 | 3.7 | Valle del Cauca | Buenaventura | medium | high |
| Regional_mammalogist | -74.626962 | 5.08294 | Cundinamaca | Guaduas | medium | high |
| Regional_mammalogist | -73.232617 | 4.222003 | Meta | Cumaral | medium | high |
| Regional_mammalogist | -73.19245 | 7.069599 | Santander | Giron | medium | high |
| Regional_mammalogist | -76.107123 | 5.207073 | Risaralda | Pueblo Rico | medium | high |
| Regional_mammalogist | -78.753074 | 1.795281 | Nariño | San Andres de Tumaco | medium | high |
| Regional_mammalogist | -74.147039 | 11.25197 | Magdalena | Santa Marta | medium | high |
| Regional_mammalogist | -74.728403 | 11.021874 | Magdalena | Sitionuevo | medium | low |
| Regional_mammalogist | -72.41554 | 5.352458 | Casanare | Yopal | medium | high |
| Regional_mammalogist | -73.955107 | 2.068044 | Meta | La Macarena | medium | high |
| Regional_mammalogist | -69.955081 | -4.190238 | Amazonas | Leticia | medium | high |
| Regional_mammalogist | -74.351229 | 4.625207 | Cundinamaca | San Antonio del Tequendama | medium | high |
| Regional_mammalogist | -75.344485 | 10.155416 | Bolivar | Arjona | medium | high |
| Regional_mammalogist | -74.979526 | 6.687047 | Antioquia | Yolombo | medium | high |
| Regional_mammalogist | -74.67688 | 4.063931 | Tolima | Cunday | medium | high |
| Regional_mammalogist | -74.760388 | 8.111908 | Antioquia | Nechi | medium | high |
| Regional_mammalogist | -75.404166 | 7.293947 | Antioquia | Valdivia | medium | high |
| Regional_mammalogist | -76.614355 | 2.470912 | Cauca | Popayan | medium | high |
| Regional_mammalogist | -75.542162 | 1.766959 | Caqueta | Florencia | medium | high |
| Regional_mammalogist | -72.670139 | 2.579603 | Guaviare | San Jose del Guaviare | medium | high |
| Regional_mammalogist | -75.804007 | 9.20312 | Cordoba | Lorica | medium | high |
| Regional_mammalogist | -74.598448 | 4.320114 | Cundinamaca | Nilo | medium | high |
| Regional_mammalogist | -73.741421 | 4.150324 | Meta | Acacias | medium | high |
| Regional_mammalogist | -68.258 | 3.919 | Guainia | Inirida | medium | high |
| Regional_mammalogist | -73.941074 | 6.280645 | Santander | Cimitarra | medium | high |
| Regional_mammalogist | -74.059489 | 3.378732 | Meta | Mesetas | medium | high |
| Regional_mammalogist | -73.74541 | 3.042187 | Meta | Vistahermosa | medium | high |
| Regional_mammalogist | -73.128726 | 7.512836 | Santander | El Playon | medium | high |
| Regional_mammalogist | -76.773073 | 2.75169 | Cauca | Morales | medium | high |
| Regional_mammalogist | -74.628056 | 2.758056 | Meta | Uribe | medium | high |
| Regional_mammalogist | -74.625703 | 2.703248 | Meta | Uribe | medium | high |
| Literature_review | -74.807466 | 0.131819 | Putumayo | Puerto Leguizamo | high | low |
| Literature_review | -73.790798 | 7.457211 | Santander | Puerto Wilches | high | high |
| Literature_review | -74.211631 | 11.138417 | Magdalena | Santa Marta | high | high |
| Literature_review | -75.545946 | 10.060202 | Sucre | San Onofre | medium | low |
| Literature_review | -71.386302 | 1.891414 | Guaviare | El Retorno | medium | medium |
| Literature_review | -74.45 | 10.95 | Magdalena | Puebloviejo | low | medium |
| Literature_review | -69.587271 | -1.050407 | Vaupes | Taraira | medium | low |
| Literature_review | -75.021741 | 8.51974 | Sucre | San Marcos | low | low |
| Literature_review | -74.777272 | 8.780709 | Sucre | Sucre | low | low |
| Literature_review | -74.635743 | 8.445745 | Sucre | Guaranda | medium | low |
| Literature_review | -74.803628 | -0.188807 | Putumayo | Puerto Leguizamo | medium | low |
| Literature_review | -77.212709 | 6.048539 | Choco | Bojaya | medium | medium |
| Literature_review | -74.138619 | 2.557991 | Meta | Uribe | medium | low |
| Literature_review | -72.949273 | 1.032553 | Guaviare | Calamar | low | low |
| Literature_review | -77.264701 | 6.605454 | Choco | Bojaya | medium | medium |
| Literature_review | -70.25571 | -3.130992 | Amazonas | Tarapaca | medium | low |
| Literature_review | -73.778904 | 6.922189 | Santander | Barrancabermeja | medium | low |
| Literature_review | -73.592956 | 10.44881 | Cesar | Pueblo Bello | medium | medium |
| Literature_review | -73.937628 | 10.733917 | Magdalena | Aracataca | medium | medium |
| Literature_review | -74.979522 | 7.065057 | Antioquia | Amalfi | medium | medium |
| Literature_review | -77.225288 | 8.324473 | Choco | Acandi | medium | low |
| Literature_review | -75.784425 | 2.371715 | HUILA | Paicol | medium | medium |
| Literature_review | -74.6930556 | 7.35388889 | Antioquia | Zaragoza | medium | medium |
| Literature_review | -75.823322 | 5.468445 | Caldas | Riosucio | high | high |
| Literature_review | -75.64167 | 7.636667 | Cordoba | San Jose de Ure | medium | medium |
| Regional_mammalogist | -75.006215 | 5.488787 | Caldas | Samana | medium | high |
| Regional_mammalogist | -74.707554 | 5.454004 | Caldas | La Dorada | medium | high |
| Literature_review | -74.6433333 | 7.32319444 | Antioquia | Segovia | high | high |
| Literature_review | -69.177885 | 2.036263 | Guainia | Pana Pana | low | low |
| Literature_review | -71.899617 | 1.82393 | Guaviare | Miraflores | low | low |
| Literature_review | -70.513276 | 4.837122 | Vichada | Santa Rosalia | medium | low |
| Regional_mammalogist | -74.1667 | 2.6667 | Meta | Uribe | medium | high |
| Regional_mammalogist | -75.397264 | 7.640748 | Antioquia | Taraza | medium | high |
| Regional_mammalogist | -74.760549 | 6.390664 | Antioquia | Caracoli | medium | high |
| Regional_mammalogist | -73.183273 | 9.043021 | Norte de Santander | Convencion | medium | high |
| Regional_mammalogist | -75.59 | 0.349 | Putumayo | Puerto Leguizamo | medium | high |
| Regional_mammalogist | -76.933333 | 3.683333 | Valle del Cauca | Buenaventura | medium | high |
| Literature_review | -70.704648 | 4.723113 | Vichada | Cumaribo | low | low |
| Literature_review | -70.448943 | 5.20765 | Vichada | La Primavera | medium | low |
| Literature_review | -75.96667 | 7.984444 | Cordoba | Montelibano | high | medium |
| Literature_review | -76.08389 | 8.003889 | Cordoba | Tierralta | high | high |
| Regional_mammalogist | -74.67 | 7.3347 | Antioquia | Segovia | medium | high |
| Regional_mammalogist | -70.139563 | -3.352117 | Amazonas | Leticia | medium | high |
| Regional_mammalogist | -71.224 | -1.553 | Amazonas | Puerto Arica | medium | high |
| Regional_mammalogist | -69.992798 | -1.917657 | Amazonas | La Pedrera | medium | high |
| Regional_mammalogist | -71.20768 | 1.948879 | Guaviare | El Retorno | medium | high |
| Regional_mammalogist | -73.755492 | 2.376729 | Meta | La Macarena | medium | high |
| Regional_mammalogist | -71.887 | -0.836 | Amazonas | Puerto Santander | medium | high |
| Regional_mammalogist | -76.141101 | 7.524984 | Cordoba | Tierralta | medium | high |
| Regional_mammalogist | -74.686883 | 0.884844 | Caqueta | Cartage de Chaira | medium | high |
| Regional_mammalogist | -72.252 | -0.148 | Caqueta | Solano | medium | high |
| Regional_mammalogist | -72.872095 | 0.757 | Caqueta | Solano | medium | high |
| Regional_mammalogist | -77.080843 | 7.749666 | Antioquia | Turbo | medium | high |
| Regional_mammalogist | -69.066494 | 2.399347 | Guainia | Puerto Colombia | medium | high |
| Regional_mammalogist | -70.110995 | 1.837362 | Guainia | Pana Pana | medium | high |
| Literature_review | -67.91464 | 6.201567 | Vichada | Puerto Carreño | low | low |
| Literature_review | -69.152449 | 5.114412 | Vichada | Cumaribo | medium | low |
| Literature_review | -68.386181 | 4.836035 | Vichada | Cumaribo | low | low |
| Literature_review | -68.24876 | 4.842142 | Vichada | Cumaribo | low | low |
| Literature_review | -68.193791 | 5.19333 | Vichada | Cumaribo | medium | low |
| Literature_review | -68.422827 | 5.19333 | Vichada | Cumaribo | medium | low |
| Literature_review | -67.501973 | 6.136003 | Vichada | Puerto Carreño | medium | low |
| Literature_review | -69.148377 | 5.83511 | Vichada | La Primavera | medium | low |
| Literature_review | -67.877994 | 5.330214 | Vichada | Cumaribo | medium | low |
| Literature_review | -68.633539 | 5.113931 | Vichada | Cumaribo | medium | low |
| Literature_review | -68.807606 | 5.019263 | Vichada | Cumaribo | medium | low |
| Literature_review | -69.024427 | 5.41015 | Vichada | La Primavera | medium | low |
| Literature_review | -68.816768 | 5.459011 | Vichada | La Primavera | medium | low |
| Literature_review | -68.624378 | 5.565894 | Vichada | Puerto Carreño | medium | low |
| Literature_review | -68.346482 | 5.510926 | Vichada | Cumaribo | medium | low |
| Literature_review | -72.041808 | 4.9267 | Casanare | Orocue | medium | low |
| Literature_review | -70.347149 | 4.922629 | Vichada | Santa Rosalia | low | low |
| Literature_review | -69.953868 | -4.169673 | Amazonas | Leticia | medium | low |
| Literature_review | -70.310975 | -3.551975 | Amazonas | Leticia | medium | low |
| Literature_review | -69.693277 | -1.891911 | Amazonas | La Pedrera | medium | low |
| Literature_review | -70.957627 | -1.467243 | Amazonas | Puerto Arica | medium | low |
| Literature_review | -68.089446 | 6.184995 | Vichada | Puerto Carreño | medium | low |
| Literature_review | -70.521739 | 5.441925 | Vichada | La Primavera | medium | low |
| Literature_review | -69.13131 | 4.625322 | Vichada | Cumaribo | medium | low |
| Regional_mammalogist | -70.212368 | -3.760399 | Amazonas | Leticia | medium | high |
| Regional_mammalogist | -73.7642 | 11.1256 | Magdalena | Santa Marta | medium | high |
| Regional_mammalogist | -73.310197 | 9.035794 | Norte de Santander | El Carmen | medium | high |
| Regional_mammalogist | -77.14792 | 5.880272 | Choco | Alto Baudo | medium | high |
| Regional_mammalogist | -75.012945 | 5.485448 | Caldas | Samana | medium | high |
| Regional_mammalogist | -72.044451 | 4.8859 | Casanare | Orocue | medium | high |
| Regional_mammalogist | -69.178319 | 5.894895 | Vichada | La Pirmavera | medium | high |
| Regional_mammalogist | -68.1547 | 6.143488 | Vichada | Puerto Carreño | medium | high |
| Regional_mammalogist | -68.300931 | 4.871277 | Vichada | Cumaribo | medium | high |
| Regional_mammalogist | -70.509023 | 4.783538 | Vichada | Santa Rosalia | medium | high |
| Regional_mammalogist | -68.988218 | 4.330221 | Vichada | Cumaribo | medium | high |
| Regional_mammalogist | -69.149073 | 2.31223 | Guainia | Pana Pana | medium | high |
| Regional_mammalogist | -69.280681 | 1.932029 | Guainia | Pana Pana | medium | high |
| Regional_mammalogist | -73.609125 | 2.531577 | Meta | La Macarena | medium | high |
| Regional_mammalogist | -74.062442 | 3.306603 | Meta | Mesetas | medium | high |
| Regional_mammalogist | -75.627116 | 1.566451 | Caqueta | Florencia | medium | high |
| Regional_mammalogist | -74.296412 | 0.352732 | Caqueta | Cartagena del Chaira | medium | high |
| Regional_mammalogist | -73.565255 | 0.440471 | Caqueta | Cartagena del Chaira | medium | high |
| Regional_mammalogist | -72.386 | -0.896 | Amazonas | Puerto Santander | medium | high |
| Regional_mammalogist | -71.362 | -0.015 | Amazonas | La Victoria | medium | high |
| Regional_mammalogist | -71.050078 | -1.445912 | Amazonas | Puerto Arica | medium | high |
| Regional_mammalogist | -70.02646 | -2.001591 | Amazonas | La Pedrera | medium | high |
| Regional_mammalogist | -69.500027 | -1.328927 | Amazonas | La Pedrera | medium | high |
| Regional_mammalogist | -75.203045 | 0.118762 | Putumayo | Puerto Leguizamo | medium | high |
| Regional_mammalogist | -75.106512 | 7.928345 | Antioquia | Caucasia | medium | high |
| GBIF | -76.104835 | 8.000715 | Cordoba | Tierralta | high | high |
| Literature_review | -76.9202 | 6.81965 | Antioquia | Vigia del Fuerte | high | low |
| Literature_review | -67.93529 | 5.894726 | Vichada | Puerto Carreño | medium | high |
| Literature_review | -67.4730556 | 6.18777778 | Vichada | Puerto Carreño | medium | high |
| GBIF | -75.937185 | 8.069485 | Cordoba | Tierralta | high | high |
| GBIF | -76.779083 | 6.016472 | Choco | Medio Atrato | medium | high |
| Literature_review | -74.19475 | 6.72466667 | Antioquia | Yondo | high | high |
| Literature_review | -74.6158889 | 6.54683333 | Antioquia | Puerto Berrio | medium | high |
| Literature_review | -75.93718 | 8.06949 | Cordoba | Tierralta | high | low |
| Regional_mammalogist | -75.91841 | 6.848642 | Antioquia | Buritaca | medium | high |
| Literature_review | -75.996584 | 7.969514 | Cordoba | Tierralta | medium | high |
| Literature_review | -75.983656 | 8.006757 | Cordoba | Tierralta | medium | high |
| Literature_review | -75.862628 | 7.895062 | Cordoba | Montelibano | medium | medium |
| Literature_review | -75.902803 | 7.864343 | Cordoba | Montelibano | medium | medium |
| Literature_review | -76.9202 | 6.81965 | Antioquia | Vigia del Fuerte | medium | low |
| Literature_review | -78.133333 | 1.35 | Nariño | Barbacoas | medium | low |
| GBIF | -74.511382 | 6.758459 | Antioquia | Remedios | medium | medium |
| GBIF | -77.04815 | 7.991347 | Choco | Unguia | medium | medium |
| Literature_review | -74.7596111 | 6.19083333 | Antioquia | Puerto Nare | high | high |
| Regional_mammalogist | -74.4842 | 6.6836 | Antioquia | Puerto Berrio | medium | high |
| Regional_mammalogist | -73.2719 | 10.5742 | Cesar | Valledupar | high | high |
| GBIF | -76.513056 | 4.073611 | Choco | El Litoral de San Juan | medium | medium |
| Regional_mammalogist | -72.986667 | 11.184861 | La Guajira | Riohacha | high | high |
| Regional_mammalogist | -72.991 | 11.190389 | La Guajira | Riohacha | medium | high |
| Regional_mammalogist | -74.7594 | 6.1908 | Antioquia | Puerto Nare | medium | high |
| Regional_mammalogist | -72.7659583 | 8.93662778 | Norte de Santander | Tibu | medium | high |
| Regional_mammalogist | -74.8005083 | 7.93606944 | Antioquia | El Bagre | medium | high |
| Regional_mammalogist | -76.7942111 | 7.76067778 | Antioquia | Carepa | medium | high |
| Regional_mammalogist | -76.8826472 | 7.334775 | Choco | Riosucio | medium | high |
| Regional_mammalogist | -74.6167611 | 7.51936389 | Antioquia | El Bagre | medium | high |
| Regional_mammalogist | -76.7047667 | 5.936275 | Choco | Medio Atrato | medium | high |
| Regional_mammalogist | -75.7265861 | 6.52975833 | Antioquia | Sopetran | medium | high |
| Regional_mammalogist | -75.8818444 | 6.2629 | Antioquia | Anza | medium | high |
| Regional_mammalogist | -74.8976139 | 6.46166389 | Antioquia | San Roque | medium | high |
| Regional_mammalogist | -67.9108194 | 6.15914722 | Vichada | Puerto Carreño | medium | high |
| Regional_mammalogist | -68.8364528 | 5.80991389 | Vichada | La Primavera | medium | high |
| Regional_mammalogist | -75.9765194 | 5.82334167 | Antioquia | Ciudad Bolivar | medium | high |
| Regional_mammalogist | -74.9711194 | 5.37396389 | Caldas | Samana | medium | high |
| Regional_mammalogist | -68.8478417 | 5.06104722 | Vichada | Cumaribo | medium | high |
| Regional_mammalogist | -70.02 | 5.361 | Vichada | La Primavera | medium | high |
| Regional_mammalogist | -69.4020278 | 4.89401944 | Vichada | Cumaribo | medium | high |
| Regional_mammalogist | -69.609 | 4.127 | Vichada | Cumaribo | medium | high |
| Regional_mammalogist | -69.954 | 4.393 | Vichada | Cumaribo | medium | high |
| Regional_mammalogist | -69.2715361 | 4.67501944 | Vichada | Cumaribo | medium | high |
| Regional_mammalogist | -71.573 | 4.598 | Meta | Puerto Gaitan | medium | high |
| Regional_mammalogist | -72.5108944 | 4.55136944 | Casanare | Tauramena | medium | high |
| Regional_mammalogist | -72.3171222 | 4.75428611 | Casanare | Mani | medium | high |
| Regional_mammalogist | -75.0027194 | 3.97812222 | Tolima | Saldaña | medium | high |
| Regional_mammalogist | -73.9402861 | 3.34322778 | Meta | San Juan de Arama | medium | high |
| Regional_mammalogist | -72.3543167 | 2.56768333 | Guaviare | San Jose del Guaviare | medium | high |
| Regional_mammalogist | -68.8448111 | 2.25685556 | Guainia | Pana Pana | medium | high |
| Regional_mammalogist | -70.5264472 | 1.86879722 | Vaupes | Papunahua | medium | high |
| Regional_mammalogist | -74.3791917 | 1.58797778 | Caqueta | San Vicente del Caguan | medium | high |
| Regional_mammalogist | -71.4020944 | 1.45258333 | Vaupes | Caruru | medium | high |
| Regional_mammalogist | -74.7707389 | 0.12301111 | Putumayo | Puerto Leguizamo | medium | high |
| Regional_mammalogist | -73.6377611 | 0.50313333 | Caqueta | Solano | medium | high |
| Regional_mammalogist | -72.11 | 0.27 | Caqueta | Solano | medium | high |
| Regional_mammalogist | -71.2203306 | 0.62099444 | Vaupes | Caruru | medium | high |
| Regional_mammalogist | -71.5884722 | 0.65739167 | Vaupes | Pacoa | medium | high |
| Regional_mammalogist | -70.9299694 | -0.54853889 | Amazonas | Miriti - Parana | medium | high |
| Regional_mammalogist | -70.388 | -1.697 | Amazonas | La Pedrera | medium | high |
| Regional_mammalogist | -69.8802972 | -2.58099167 | Amazonas | Tarapaca | medium | high |
| Regional_mammalogist | -74.70445 | 10.148775 | Magdalena | Zapayan | medium | high |
| Regional_mammalogist | -74.4474667 | 9.96796389 | Magdalena | Plato | medium | high |
| Regional_mammalogist | -74.29 | 10.171 | Magdalena | Sabanas de San Angel | medium | high |
| Regional_mammalogist | -74.7316139 | 9.94929444 | Magdalena | Tenerife | medium | high |
| Regional_mammalogist | -74.6346528 | 10.4084972 | Magdalena | El Pinon | medium | high |
| Regional_mammalogist | -72.0544889 | 2.84602222 | Meta | Mapiripan | medium | high |
| Regional_mammalogist | -74.0829722 | 11.0845278 | Magdalena | Santa Marta | medium | high |
| Regional_mammalogist | -75.3300556 | 10.0399444 | Bolivar | Maria La Baja | medium | high |
| Regional_mammalogist | -74.0547222 | 10.5255278 | Magdalena | Aracataca | medium | high |
| Regional_mammalogist | -73.9092222 | 11.2633333 | Magdalena | Santa Marta | medium | high |
| Regional_mammalogist | -72.758677 | 11.080678 | La Guajira | Hatonuevo | medium | high |
| Regional_mammalogist | -72.83649 | 11.186732 | La Guajira | Riohacha | medium | high |
| Regional_mammalogist | -73.059334 | 11.111834 | La Guajira | Riohacha | medium | high |
| Regional_mammalogist | -73.203693 | 10.95461 | La Guajira | Riohacha | medium | high |
| Regional_mammalogist | -73.082653 | 10.836734 | La Guajira | San Juan del Cesar | medium | high |
| Regional_mammalogist | -73.116631 | 10.789101 | La Guajira | San Juan del Cesar | medium | high |
| Regional_mammalogist | -72.780148 | 10.969201 | La Guajira | Barrancas | medium | high |
| Regional_mammalogist | -72.974586 | 10.621297 | La Guajira | Villanueva | medium | high |
| Regional_mammalogist | -73.00714 | 10.574108 | La Guajira | Villanueva | medium | high |
| Regional_mammalogist | -73.111613 | 11.294602 | La Guajira | Riohacha | medium | high |
| Regional_mammalogist | -73.211008 | 11.257035 | La Guajira | Dibulla | medium | high |
| Regional_mammalogist | -73.45895 | 11.116084 | La Guajira | Dibulla | medium | high |
| Regional_mammalogist | -73.637366 | 11.114762 | La Guajira | Dibulla | medium | high |
| Regional_mammalogist | -72.901226 | 11.549074 | La Guajira | Riohacha | medium | high |
| Literature_review | -74.200389 | 11.136234 | Magdalena | Santa Marta | high | high |
| GBIF | -76.5605 | 6.699083 | Antioquia | Frontino | medium | high |
| GBIF | -73.153653 | 3.69696 | Meta | San Martin | medium | high |
| GBIF | -72.440003 | 3.537326 | Meta | San Martin | medium | high |
| GBIF | -73.457907 | 3.570986 | Meta | San Martin | medium | high |
| GBIF | -72.440003 | 3.537326 | Meta | San Martin | medium | high |
| GBIF | -72.945979 | 4.017353 | Meta | Puerto Lopez | medium | high |
| GBIF | -72.542453 | 3.824689 | Meta | Puerto Lopez | medium | high |
| GBIF | -72.162993 | 4.03722 | Meta | Puerto Lopez | medium | high |
| GBIF | -73.699844 | 4.15745 | Meta | Villavicencio | medium | high |
| GBIF | -73.69354 | 4.167199 | Meta | Villavicencio | medium | high |
| GBIF | -73.402054 | 3.531515 | Meta | San Martin | medium | high |
| GBIF | -73.402054 | 3.531515 | Meta | San Martin | medium | high |
| GBIF | -72.608264 | 3.810341 | Meta | Puerto Lopez | medium | high |
| GBIF | -72.803689 | 4.09235 | Meta | Puerto Lopez | medium | high |
| GBIF | -73.402054 | 3.531515 | Meta | San Martin | medium | high |
| GBIF | -72.445877 | 4.198196 | Meta | Puerto Lopez | medium | high |
| GBIF | -72.431705 | 4.190973 | Meta | Puerto Lopez | medium | high |
| GBIF | -71.931156 | 3.899235 | Meta | Puerto Gaitan | medium | high |
| GBIF | -73.900875 | 3.621519 | Meta | El Castillo | medium | high |
| GBIF | -71.761631 | 3.634763 | Meta | Puerto Gaitan | medium | high |
| GBIF | -71.373363 | 3.485469 | Meta | Puerto Gaitan | medium | high |
| GBIF | -71.373363 | 3.485469 | Meta | Puerto Gaitan | medium | high |
| GBIF | -73.89639 | 3.615064 | Meta | El Castillo | medium | high |
| GBIF | -71.931156 | 3.899235 | Meta | Puerto Gaitan | medium | high |
| GBIF | -71.761631 | 3.634763 | Meta | Puerto Gaitan | medium | high |
| GBIF | -71.655472 | 4.563219 | Meta | Puerto Gaitan | medium | high |
| GBIF | -73.900875 | 3.621519 | Meta | El Castillo | medium | high |
| GBIF | -71.373363 | 3.485469 | Meta | Puerto Gaitan | medium | high |
| GBIF | -71.621656 | 4.592994 | Meta | Puerto Gaitan | medium | high |
| GBIF | -73.149157 | 3.624423 | Meta | San Martin | medium | high |
| GBIF | -73.637869 | 3.679461 | Meta | San Martin | medium | high |
| GBIF | -73.425311 | 3.536348 | Meta | San Martin | medium | high |
| GBIF | -72.942968 | 3.433742 | Meta | San Martin | medium | high |
| GBIF | -73.402054 | 3.531515 | Meta | San Martin | medium | high |
| GBIF | -72.450278 | 3.50019 | Meta | San Martin | medium | high |
| GBIF | -72.627905 | 3.369002 | Meta | San Martin | medium | high |
| GBIF | -72.511963 | 3.452407 | Meta | San Martin | medium | high |
| GBIF | -72.431705 | 4.190973 | Meta | Puerto Lopez | medium | high |
| GBIF | -72.542453 | 3.824689 | Meta | Puerto Lopez | medium | high |
| GBIF | -72.936596 | 4.032437 | Meta | Puerto Lopez | medium | high |
| GBIF | -72.504239 | 3.858002 | Meta | Puerto Lopez | medium | high |
| GBIF | -72.977099 | 3.820359 | Meta | Puerto Lopez | medium | high |
| GBIF | -71.646235 | 4.578634 | Meta | Puerto Gaitan | medium | high |
| GBIF | -71.752629 | 3.563484 | Meta | Puerto Gaitan | medium | high |
| GBIF | -71.373363 | 3.485469 | Meta | Puerto Gaitan | medium | high |
| GBIF | -71.620804 | 4.606237 | Meta | Puerto Gaitan | medium | high |
| GBIF | -73.892549 | 3.619492 | Meta | El Castillo | medium | high |
| GBIF | -71.752629 | 3.563484 | Meta | Puerto Gaitan | medium | high |
| GBIF | -73.884262 | 3.608438 | Meta | El Castillo | medium | high |
| GBIF | -71.620804 | 4.606237 | Meta | Puerto Gaitan | medium | high |
| GBIF | -72.280793 | 3.756763 | Meta | Puerto Gaitan | medium | high |
| GBIF | -71.447016 | 3.560771 | Meta | Puerto Gaitan | medium | high |
| GBIF | -72.316827 | 4.280748 | Meta | Puerto Lopez | medium | high |
| GBIF | -71.52892 | 3.554069 | Meta | Puerto Gaitan | medium | high |
| GBIF | -71.52892 | 3.554069 | Meta | Puerto Gaitan | medium | high |
| GBIF | -73.912954 | 3.623849 | Meta | El Castillo | medium | high |
| GBIF | -71.752629 | 3.563484 | Meta | Puerto Gaitan | medium | high |
| GBIF | -73.892762 | 3.607218 | Meta | El Castillo | medium | high |
| GBIF | -73.88747 | 3.606216 | Meta | El Castillo | medium | high |
| GBIF | -73.91263 | 3.645929 | Meta | El Castillo | medium | high |
| GBIF | -71.52892 | 3.554069 | Meta | Puerto Gaitan | medium | high |
| GBIF | -77.271111 | 6.092778 | Choco | Bojaya | medium | medium |
| Literature_review | -74.4997778 | 6.66783333 | Antioquia | Puerto Berrio | high | high |
| Literature_review | -74.4997778 | 6.66786111 | Antioquia | Puerto Berrio | high | high |
| Regional_mammalogist | -74.4861 | 6.9647 | Antioquia | Remedios | medium | high |
| Regional_mammalogist | -71.3764 | 6.0328 | Casanare | Hato Corozal | medium | high |
| Regional_mammalogist | -73.501363 | 6.824963 | Santander | San Vicente de Chucuri | medium | high |
| Regional_mammalogist | -74.1953 | 6.75 | Antioquia | Yondo | medium | high |
| Literature_review | -74.583333 | 7.333333 | Antioquia | Segovia | high | medium |
| Literature_review | -75.100365 | 7.238759 | Antioquia | Anori | high | medium |
| Literature_review | -75.138289 | 7.009061 | Antioquia | Anori | high | medium |
| Literature_review | -74.28497 | 6.685668 | Antioquia | Yondo | high | high |
| Literature_review | -74.499778 | 6.667861 | Antioquia | Puerto Berrio | high | medium |
| Literature_review | -74.5211 | 6.9658 | Antioquia | Remedios | high | medium |
| GBIF | -74.096389 | 7.738111 | Bolivar | Simiti | high | low |
| GBIF | -74.283611 | 7.795444 | Bolivar | Santa Rosa del Sura | high | low |
| GBIF | -74.093528 | 7.743722 | Bolivar | Simiti | high | low |
| GBIF | -74.093528 | 7.743722 | Bolivar | Simiti | high | low |
| GBIF | -73.772222 | 7.498889 | Santander | Sabana de Torres | medium | high |
| GBIF | -73.339722 | 7.421667 | Santander | Rionegro | medium | medium |
| Literature_review | -73.33972 | 7.42167 | Santander | Rionegro | high | low |
| Literature_review | -73.77222 | 7.49889 | Santander | Sabana de Torres | high | low |
| Regional_mammalogist | -74.121 | 11.296944 | Magdalena | Santa Marta | high | high |
| Regional_mammalogist | -74.002444 | 11.319861 | Magdalena | Santa Marta | high | high |
| Regional_mammalogist | -74.045833 | 11.297028 | Magdalena | Santa Marta | high | high |
| Regional_mammalogist | -73.968694 | 11.325889 | Magdalena | Santa Marta | high | high |
| Regional_mammalogist | -74.203778 | 11.145083 | Magdalena | Santa Marta | high | high |
| Regional_mammalogist | -74.201611 | 11.143139 | Magdalena | Santa Marta | high | high |
| Regional_mammalogist | -74.206278 | 11.141111 | Magdalena | Santa Marta | high | high |
| Regional_mammalogist | -74.085111 | 11.315389 | Magdalena | Santa Marta | medium | high |
| Regional_mammalogist | -74.036833 | 11.319222 | Magdalena | Santa Marta | medium | high |
| Regional_mammalogist | -74.044472 | 11.292528 | Magdalena | Santa Marta | medium | high |
| Literature_review | -73.875715 | 11.242712 | Magdalena | Santa Marta | high | medium |
| GBIF | -77.346278 | 2.770167 | Cauca | Timbiqui | medium | high |
| GBIF | -77.122222 | 7.437778 | Choco | Riosucio | medium | high |
| GBIF | -77.248056 | 5.6805 | Choco | Nuqui | medium | high |
| Literature_review | -74.6356194 | 8.70525 | Sucre | Majagual | high | high |
| Literature_review | -74.6644 | 8.76727778 | Sucre | Sucre | medium | high |
| Literature_review | -74.63781 | 8.705255 | Sucre | Majagual | high | high |
| GBIF | -76.131647 | 4.845017 | Valle del Cauca | El Aguila | medium | high |
| Literature_review | -76.5990556 | 5.68488889 | Choco | Quibdo | high | high |
| GBIF | -71.604336 | 6.1995 | Casanare | Hato Corozal | medium | high |
| GBIF | -69.76345 | 6.433333 | Arauca | Cravo Norte | medium | high |
| GBIF | -76.973044 | 6.932186 | Choco | Carmen del Darien | medium | medium |
| GBIF | -76.973044 | 6.932186 | Choco | Carmen del Darien | medium | medium |
| GBIF | -76.973044 | 6.932186 | Choco | Carmen del Darien | medium | medium |
| GBIF | -74.347139 | 7.12825 | Bolivar | San Pablo | high | high |
| GBIF | -74.347889 | 7.143389 | Bolivar | San Pablo | high | high |
| GBIF | -74.347139 | 7.12825 | Bolivar | San Pablo | high | high |
| GBIF | -74.327028 | 7.684222 | Bolivar | Santa Rosa del Sura | high | high |
| GBIF | -71.4924 | 5.935867 | Casanare | Hato Corozal | medium | high |
| GBIF | -71.566 | 6.00765 | Casanare | Hato Corozal | medium | high |
| GBIF | -71.466817 | 5.922683 | Casanare | Hato Corozal | medium | high |
| GBIF | -71.488517 | 5.931117 | Casanare | Hato Corozal | medium | high |
| Literature_review | -71.46682 | 5.92268 | Casanare | Hato Corozal | high | low |
| Literature_review | -71.48852 | 5.93112 | Casanare | Hato Corozal | high | low |
| Literature_review | -71.4924 | 5.93587 | Casanare | Hato Corozal | high | low |
| Literature_review | -71.566 | 6.00765 | Casanare | Hato Corozal | high | low |
| Literature_review | -74.18333 | 6.797056 | Antioquia | Yondo | high | high |
| Literature_review | -74.27356 | 6.77536 | Antioquia | Yondo | high | high |
| Literature_review | -74.15592 | 6.69558 | Santander | Cimitarra | high | high |
| Literature_review | -74.27069 | 6.769583 | Antioquia | Yondo | high | high |
| Literature_review | -74.20986 | 6.765222 | Antioquia | Yondo | high | high |
| Literature_review | -74.14031 | 6.72425 | Santander | Cimitarra | high | low |
| Literature_review | -74.337 | 6.72544 | Antioquia | Yondo | high | high |
| Literature_review | -74.37444 | 6.6955 | Antioquia | Yondo | high | high |
| Literature_review | -74.37478 | 6.70192 | Antioquia | Yondo | high | high |
| Literature_review | -74.34597 | 6.73075 | Antioquia | Yondo | high | high |
| SIB | -71.188567 | 6.550333 | Arauca | Arauquita | medium | medium |
| Regional_mammalogist | -74.6414444 | 0.16647222 | Caqueta | Solano | high | high |
| Regional_mammalogist | -74.7561 | 0.43676667 | Caqueta | Solano | high | high |
| Regional_mammalogist | -74.7395917 | 0.43740278 | Caqueta | Solano | medium | high |
| Regional_mammalogist | -71.79225 | 0.30151111 | Caqueta | Solano | medium | high |
| Regional_mammalogist | -72.1279 | 0.55443056 | Caqueta | Solano | medium | high |
| Regional_mammalogist | -73.9975278 | -0.99152778 | Amazonas | Puerto Alegria | medium | high |
| GBIF | -74.365018 | 6.741252 | Antioquia | Yondo | medium | medium |
| GBIF | -73.793 | 2.261 | Meta | La Macarena | medium | high |
| GBIF | -77.171417 | 7.412861 | Choco | Riosucio | medium | high |
| GBIF | -77.171417 | 7.412861 | Choco | Riosucio | medium | high |
| Literature_review | -74.21159 | 6.75629 | Antioquia | Yondo | high | low |
| SIB | -72.543312 | 11.197674 | La Guajira | Albania | high | medium |
| GBIF | -70.7744 | 5.3304 | Casanare | Trinidad | high | high |
| GBIF | -67.906255 | 3.885202 | Guainia | Inirida | medium | medium |
| GBIF | -75.904056 | 1.685222 | Caqueta | Belen de los Andaquies | high | high |
| GBIF | -74.297458 | 6.916236 | Antioquia | Yondo | medium | medium |
| GBIF | -74.37098 | 6.94432 | Antioquia | Remedios | medium | medium |
| GBIF | -74.274346 | 6.809912 | Antioquia | Yondo | medium | medium |
| GBIF | -74.249129 | 6.842582 | Antioquia | Yondo | medium | medium |
| GBIF | -74.310663 | 6.84956 | Antioquia | Yondo | medium | medium |
| GBIF | -74.223053 | 6.857086 | Antioquia | Yondo | medium | medium |
| GBIF | -70.198633 | 6.71205 | Arauca | Arauca | medium | high |
| GBIF | -74.26966 | 6.827276 | Antioquia | Yondo | medium | medium |
| Literature_review | -73.21291 | 9.46466 | Cesar | La Jagua de Ibirico | medium | high |
| Literature_review | -73.14594 | 9.54998 | Cesar | La Jagua de Ibirico | medium | high |
| Literature_review | -68.16044 | 5.93047 | Vichada | Puerto Carreño | high | high |
| Literature_review | -74.21159 | 6.75629 | Antioquia | Yondo | high | low |
| Literature_review | -74.15592 | 6.69555 | Santander | Cimitarra | high | high |
| Literature_review | -67.88197 | 6.09069 | Vichada | Puerto Carreño | high | high |
| Literature_review | -74.33699 | 6.72539 | Antioquia | Yondo | high | high |
| Literature_review | -74.23656 | 6.79718 | Antioquia | Yondo | high | high |
| Literature_review | -74.37452 | 6.69543 | Antioquia | Yondo | high | high |
| Literature_review | -74.35062 | 6.71641 | Antioquia | Yondo | high | high |
| Literature_review | -74.24663 | 6.81982 | Antioquia | Yondo | high | high |
| Literature_review | -74.37175 | 6.77832 | Antioquia | Yondo | high | high |
| SIB | -72.599816 | 11.106259 | La Guajira | Barrancas | high | medium |
| SIB | -73.72536 | 7.039264 | Santander | Barrancabermeja | high | medium |
| SIB | -73.654929 | 7.061005 | Santander | Barrancabermeja | high | medium |
| SIB | -73.603126 | 7.111734 | Santander | Barrancabermeja | high | medium |
| SIB | -73.742574 | 7.057154 | Santander | Barrancabermeja | high | medium |
| SIB | -73.607943 | 7.016607 | Santander | San Vicente de Chucuri | high | medium |
| SIB | -73.725325 | 7.152677 | Santander | Barrancabermeja | high | medium |
| SIB | -73.892662 | 7.195467 | Santander | PUERTO WILCHES | high | medium |
| SIB | -73.873053 | 7.061007 | Santander | Barrancabermeja | high | medium |
| GBIF | -74.228015 | 6.993627 | Antioquia | Yondo | medium | medium |
| GBIF | -70.787 | 5.3387 | Casanare | Trinidad | high | high |
| GBIF | -70.8018 | 5.3131 | Casanare | Trinidad | high | high |
| GBIF | -72.637389 | 1.289667 | Guaviare | Miraflores | medium | high |
| GBIF | -70.965389 | 0.123694 | Vaupes | Pacoa | medium | high |
| GBIF | -74.67272 | 6.096083 | Antioquia | Puerto Nare | medium | medium |
| GBIF | -74.200251 | 6.994688 | Antioquia | Yondo | medium | medium |
| GBIF | -74.244698 | 6.90832 | Antioquia | Yondo | medium | medium |
| GBIF | -74.268748 | 6.908599 | Antioquia | Yondo | medium | medium |
| SIB | -72.559809 | 11.178994 | La Guajira | Albania | high | medium |
| SIB | -72.630574 | 11.076478 | La Guajira | Barrancas | medium | medium |
| SIB | -72.774796 | 11.160113 | La Guajira | Riohacha | high | medium |
| SIB | -77.173658 | 7.813262 | Choco | Riosucio | medium | high |
| Regional_mammalogist | -74.6299167 | 0.25325 | Caqueta | Solano | high | high |
| Regional_mammalogist | -74.6140278 | 0.25227778 | Caqueta | Solano | high | high |
| Regional_mammalogist | -74.62874 | 0.18195 | Caqueta | Solano | high | high |
| Regional_mammalogist | -74.74957 | 0.35778 | Caqueta | Solano | high | high |
| Regional_mammalogist | -74.72719 | 0.36533 | Caqueta | Solano | high | high |
| Regional_mammalogist | -74.7161667 | 0.30972222 | Caqueta | Solano | high | high |
| Regional_mammalogist | -74.686833 | 0.327222 | Caqueta | Solano | high | high |
| Regional_mammalogist | -74.74957 | 0.35778 | Caqueta | Solano | high | high |
| Regional_mammalogist | -74.73943 | 0.36404 | Caqueta | Solano | high | high |
| Regional_mammalogist | -74.706167 | 0.327528 | Caqueta | Solano | high | high |
| Regional_mammalogist | -74.64575 | 0.29802778 | Caqueta | Solano | high | high |
| Regional_mammalogist | -74.62096 | 0.28856 | Caqueta | Solano | high | high |
| Regional_mammalogist | -74.6384 | 0.27201 | Caqueta | Solano | high | high |
| Regional_mammalogist | -74.61126 | 0.21998 | Caqueta | Solano | high | high |
| Regional_mammalogist | -74.5935 | 0.21691667 | Caqueta | Solano | high | high |
| Regional_mammalogist | -74.57635 | 0.18253 | Caqueta | Solano | high | high |
| GBIF | -77.07254 | 0.45157 | Putumayo | Valle del Guamez | high | high |
| GBIF | -74.275667 | 5.946528 | Boyaca | Puerto Boyaca | high | high |
| GBIF | -74.278167 | 5.948944 | Boyaca | Puerto Boyaca | high | high |
| Literature_review | -73.87766 | 10.04865 | Cesar | Bosconia | medium | high |
| Literature_review | -73.71833 | 10.072972 | Cesar | Valledupar | high | high |
| Literature_review | -73.755719 | 10.029653 | Cesar | Valledupar | high | high |
| Literature_review | -73.1272 | 9.56019 | Cesar | La Jagua de Ibirico | high | high |
| SIB | -72.482872 | 11.149766 | La Guajira | Maicao | high | medium |
| SIB | -72.577162 | 11.108637 | La Guajira | Albania | high | medium |
| GBIF | -72.625495 | 11.034447 | La Guajira | Barrancas | medium | medium |
| Literature_review | -73.258 | 10.165 | Cesar | San Diego | high | high |
