# Supplementary material for: Preserving the spots: Jaguar (Panthera onca) distribution and priority conservation areas in Colombia
Source: PLoS One. 2024 Mar 22;19(3):e0300375. doi: 10.1371/journal.pone.0300375 (PMC10959345; doi:10.1371/journal.pone.0300375)
Supplement: S2 Table — (DOCX) [file pone.0300375.s002.docx]

# Preserving the spots: Jaguar (*Panthera onca*) distribution and priority conservation areas in Colombia

María Camila Machado-Aguilera, Leonardo Lemus-Mejía, Jairo Pérez-Torres, Diego A. Zárrate-Charry, Andrés Arias-Alzate, José F. González-Maya.

**SUPPORTING INFORMATION**

**S2 TABLE**

**Supporting Information 2 (S2 Table).** Models constructed for species distribution modeling and corresponding parameters for *Panthera onca* in Colombia.

| **Settings** | **Features** | **Rm** | **Train**  **AUC** | **Avg.**  **Test.AUC** | **Var.**  **Test.AUC** | **Avg.**  **Diff.AUC** | **Var.**  **Diff.AUC** | **Avg.**  **Test.orMTP** | **Var.**  **Test.orMTP** | **Avg.**  **Test.or10pct** | **Var.**  **Test.or10pct** | **AICc** | **Delta.**  **AICc** | **w.AIC** | **Parameters** |
| --- | --- | --- | --- | --- | --- | --- | --- | --- | --- | --- | --- | --- | --- | --- | --- |
| LQHP_4 | LQHP | 4 | 0.6882 | 0.675073 | 0.001065 | 0.015755 | 0.00148 | 0 | 0 | 0.109106 | 0.001217 | 10753.58 | 0 | 0.534737 | 20 |
| LQHP_3 | LQHP | 3 | 0.7032 | 0.683772 | 0.00122 | 0.017152 | 0.001885 | 0.004996 | 3.45E-05 | 0.119504 | 0.000859 | 10754.05 | 0.468415 | 0.423083 | 36 |
| LQHP_3.5 | LQHP | 3.5 | 0.6952 | 0.679337 | 0.001213 | 0.016358 | 0.001668 | 0 | 0 | 0.101269 | 0.00115 | 10759.25 | 5.663645 | 0.031499 | 30 |
| LQHP_4.5 | LQHP | 4.5 | 0.6826 | 0.670919 | 0.000943 | 0.01555 | 0.001271 | 0 | 0 | 0.103929 | 0.000853 | 10762.62 | 9.039065 | 0.005825 | 17 |
| LQHP_2 | LQHP | 2 | 0.7204 | 0.699248 | 0.001107 | 0.019554 | 0.002466 | 0.002841 | 3.23E-05 | 0.12562 | 0.001963 | 10763.18 | 9.592332 | 0.004418 | 56 |
| LQHP_2.5 | LQHP | 2.5 | 0.7115 | 0.690519 | 0.001242 | 0.018764 | 0.002308 | 0.004996 | 3.45E-05 | 0.113725 | 0.002233 | 10767.84 | 14.25724 | 0.000429 | 49 |
| LQ_0.5 | LQ | 0.5 | 0.6779 | 0.671085 | 0.000581 | 0.010111 | 0.000811 | 0.004996 | 3.45E-05 | 0.113499 | 0.001147 | 10776.97 | 23.38885 | 4.46E-06 | 11 |
| LQHP_1.5 | LQHP | 1.5 | 0.7318 | 0.704599 | 0.000866 | 0.026842 | 0.002285 | 0.002841 | 3.23E-05 | 0.120805 | 0.002691 | 10778.29 | 24.70681 | 2.31E-06 | 72 |
| LQ_1 | LQ | 1 | 0.6762 | 0.670537 | 0.000558 | 0.00976 | 0.000839 | 0.002155 | 1.86E-05 | 0.113681 | 0.00169 | 10779.57 | 25.99058 | 1.21E-06 | 10 |
| LQ_1.5 | LQ | 1.5 | 0.6753 | 0.669644 | 0.000531 | 0.009947 | 0.000813 | 0.002155 | 1.86E-05 | 0.118677 | 0.001467 | 10780.71 | 27.1286 | 6.87E-07 | 8 |
| LQ_2 | LQ | 2 | 0.6748 | 0.668875 | 0.000488 | 0.01023 | 0.000732 | 0.002155 | 1.86E-05 | 0.114366 | 0.002014 | 10784.24 | 30.65422 | 1.18E-07 | 7 |
| L_0.5 | L | 0.5 | 0.6726 | 0.669076 | 0.001029 | 0.011595 | 0.001146 | 0.002155 | 1.86E-05 | 0.10744 | 0.001955 | 10786.16 | 32.57863 | 4.51E-08 | 7 |
| L_1 | L | 1 | 0.6711 | 0.667873 | 0.000948 | 0.011289 | 0.001037 | 0.002155 | 1.86E-05 | 0.111931 | 0.001983 | 10788.86 | 35.27769 | 1.17E-08 | 7 |
| LQ_2.5 | LQ | 2.5 | 0.674 | 0.667622 | 0.000451 | 0.010176 | 0.000668 | 0.002155 | 1.86E-05 | 0.11203 | 0.0017 | 10789.69 | 36.10925 | 7.71E-09 | 7 |
| L_1.5 | L | 1.5 | 0.6694 | 0.66582 | 0.000864 | 0.011197 | 0.000929 | 0.002155 | 1.86E-05 | 0.109595 | 0.001654 | 10793.27 | 39.68312 | 1.29E-09 | 7 |
| LQ_3 | LQ | 3 | 0.672 | 0.664878 | 0.000436 | 0.010274 | 0.000608 | 0.002155 | 1.86E-05 | 0.11203 | 0.0017 | 10798.31 | 44.72703 | 1.04E-10 | 7 |
| L_2 | L | 2 | 0.6671 | 0.662765 | 0.000781 | 0.011355 | 0.000816 | 0.002155 | 1.86E-05 | 0.112436 | 0.001631 | 10799.03 | 45.44402 | 7.25E-11 | 7 |
| LQH_4 | LQH | 4 | 0.6813 | 0.666262 | 0.000292 | 0.013192 | 0.000652 | 0.002841 | 3.23E-05 | 0.107916 | 0.000991 | 10800.38 | 46.79291 | 3.69E-11 | 25 |
| LQH_4.5 | LQH | 4.5 | 0.6761 | 0.655395 | 0.000162 | 0.013566 | 0.0005 | 0.002841 | 3.23E-05 | 0.110253 | 0.001272 | 10803.9 | 50.31754 | 6.34E-12 | 21 |
| L_2.5 | L | 2.5 | 0.6635 | 0.658813 | 0.00072 | 0.011442 | 0.000727 | 0.002155 | 1.86E-05 | 0.114366 | 0.002014 | 10806.51 | 52.92271 | 1.72E-12 | 7 |
| LQ_3.5 | LQ | 3.5 | 0.6677 | 0.659634 | 0.000446 | 0.011268 | 0.000586 | 0.002155 | 1.86E-05 | 0.115836 | 0.001452 | 10806.87 | 53.28544 | 1.44E-12 | 7 |
| L_3 | L | 3 | 0.6588 | 0.655069 | 0.000633 | 0.011371 | 0.000643 | 0.002155 | 1.86E-05 | 0.108783 | 0.001813 | 10812.7 | 59.11188 | 7.8E-14 | 6 |
| LQH_3.5 | LQH | 3.5 | 0.6884 | 0.672929 | 0.000396 | 0.014403 | 0.000747 | 0.002841 | 3.23E-05 | 0.111163 | 0.000886 | 10813.41 | 59.83132 | 5.44E-14 | 37 |
| LQH_2.5 | LQH | 2.5 | 0.7104 | 0.684456 | 0.000586 | 0.021164 | 0.001017 | 0.004996 | 3.45E-05 | 0.110785 | 0.001743 | 10815.95 | 62.36836 | 1.53E-14 | 56 |
| L_3.5 | L | 3.5 | 0.6577 | 0.652291 | 0.000493 | 0.011653 | 0.000606 | 0.002155 | 1.86E-05 | 0.105942 | 0.001722 | 10816.56 | 62.97473 | 1.13E-14 | 6 |
| LQ_4 | LQ | 4 | 0.6605 | 0.652133 | 0.000505 | 0.011847 | 0.000541 | 0.002155 | 1.86E-05 | 0.116746 | 0.001106 | 10817.28 | 63.69986 | 7.87E-15 | 7 |
| LQH_3 | LQH | 3 | 0.6983 | 0.678461 | 0.000498 | 0.016114 | 0.000923 | 0.002841 | 3.23E-05 | 0.11634 | 0.00118 | 10817.68 | 64.1014 | 6.44E-15 | 47 |
| L_4 | L | 4 | 0.6563 | 0.650017 | 0.000425 | 0.012029 | 0.000591 | 0.002155 | 1.86E-05 | 0.115654 | 0.000959 | 10821.03 | 67.44505 | 1.21E-15 | 6 |
| LQ_4.5 | LQ | 4.5 | 0.6541 | 0.646747 | 0.000378 | 0.011956 | 0.000462 | 0.002155 | 1.86E-05 | 0.113318 | 0.000712 | 10824.63 | 71.04184 | 2E-16 | 6 |
| L_4.5 | L | 4.5 | 0.6549 | 0.646062 | 0.000352 | 0.012236 | 0.000583 | 0.002155 | 1.86E-05 | 0.111344 | 0.001372 | 10826.1 | 72.51725 | 9.58E-17 | 6 |
| H_4 | H | 4 | 0.6907 | 0.66018 | 0.000238 | 0.017809 | 0.000474 | 0.002841 | 3.23E-05 | 0.116845 | 0.001053 | 10829.58 | 75.9965 | 1.68E-17 | 29 |
| H_3.5 | H | 3.5 | 0.7019 | 0.670773 | 0.000462 | 0.02206 | 0.000626 | 0.002841 | 3.23E-05 | 0.108421 | 0.000847 | 10833.17 | 79.58484 | 2.8E-18 | 39 |
| H_2 | H | 2 | 0.7304 | 0.705551 | 0.000422 | 0.02435 | 0.000868 | 0.002841 | 3.23E-05 | 0.122751 | 0.000766 | 10842.15 | 88.56895 | 3.13E-20 | 75 |
| LQH_1.5 | LQH | 1.5 | 0.7319 | 0.70131 | 0.000495 | 0.030318 | 0.001375 | 0.002841 | 3.23E-05 | 0.126865 | 0.001638 | 10842.39 | 88.80231 | 2.79E-20 | 86 |
| LQH_2 | LQH | 2 | 0.7214 | 0.69516 | 0.000501 | 0.022937 | 0.001174 | 0.004996 | 3.45E-05 | 0.123157 | 0.000625 | 10844.25 | 90.66315 | 1.1E-20 | 76 |
| H_4.5 | H | 4.5 | 0.6756 | 0.652799 | 0.000219 | 0.01318 | 0.000143 | 0.002841 | 3.23E-05 | 0.111443 | 0.001433 | 10845.19 | 91.61124 | 6.84E-21 | 27 |
| H_3 | H | 3 | 0.7148 | 0.687274 | 0.000289 | 0.022688 | 0.000657 | 0.002841 | 3.23E-05 | 0.111667 | 0.000738 | 10850.93 | 97.3493 | 3.88E-22 | 57 |
| LQHP_1 | LQHP | 1 | 0.7456 | 0.710978 | 0.000456 | 0.037831 | 0.001659 | 0.002841 | 3.23E-05 | 0.136743 | 0.003979 | 10851.45 | 97.86321 | 3E-22 | 107 |
| H_1.5 | H | 1.5 | 0.736 | 0.706682 | 0.000417 | 0.030237 | 0.001192 | 0.002841 | 3.23E-05 | 0.126278 | 0.001535 | 10855.93 | 102.3439 | 3.19E-23 | 90 |
| LQH_1 | LQH | 1 | 0.7447 | 0.705131 | 0.000583 | 0.040735 | 0.001933 | 0.002841 | 3.23E-05 | 0.132085 | 0.001005 | 10869.39 | 115.8051 | 3.81E-26 | 108 |
| H_2.5 | H | 2.5 | 0.7224 | 0.698661 | 0.000373 | 0.02282 | 0.000708 | 0.002841 | 3.23E-05 | 0.111667 | 0.000738 | 10878.79 | 125.2103 | 3.46E-28 | 77 |
| LQHP_0.5 | LQHP | 0.5 | 0.7656 | 0.719459 | 0.00113 | 0.048749 | 0.002803 | 0.00985 | 0.000176 | 0.141207 | 0.002374 | 10900.91 | 147.3219 | 5.47E-33 | 138 |
| H_1 | H | 1 | 0.7455 | 0.707008 | 0.000688 | 0.039993 | 0.00209 | 0.002841 | 3.23E-05 | 0.12828 | 0.001839 | 10908.65 | 155.0684 | 1.14E-34 | 118 |
| H_0.5 | H | 0.5 | 0.762 | 0.715702 | 0.001245 | 0.049548 | 0.002541 | 0.005177 | 3.64E-05 | 0.124402 | 0.000569 | 11003.2 | 249.612 | 3.35E-55 | 155 |
| LQH_0.5 | LQH | 0.5 | 0.7621 | 0.71608 | 0.001257 | 0.04923 | 0.002532 | 0.005177 | 3.64E-05 | 0.128334 | 0.000868 | 11022.22 | 268.6366 | 2.48E-59 | 158 |
