# Supplementary material for: Preserving the spots: Jaguar (Panthera onca) distribution and priority conservation areas in Colombia
Source: PLoS One. 2024 Mar 22;19(3):e0300375. doi: 10.1371/journal.pone.0300375 (PMC10959345; doi:10.1371/journal.pone.0300375)
Supplement: Attachment — Submitted filename: Rebutal_letter_Machado-Aguilera et al.doc [file pone.0300375.s003.doc]

Bogotá, Colombia, October 21, 2023

Dr.

Jonafer Estoque Miranda

**Editorial board**

PLOS One

*Ref. Second Rebuttal letter for PLOS One*

Dear Dr. Estoque,

Thanks for your email and for the comments regarding our previous submission. In the letter we responded to the previous editor, which strangely rejected the paper on the grounds of a correction that we could not find, or understand, in our manuscript. Specifically, Dr. Cristescu´s only comment was "Thank you for your submission, this is an interesting manuscript but before we can proceed with the review process one important aspect must be adjusted. Specifically, the Materials and Methods, as well as the Results section contain several references where the source was not found. Please adjust the formatting and double-check the manuscript proof before resubmission. When resubmitting the manuscript, please mention my name (Bogdan Cristescu) in the cover letter so that the manuscript can be re-assigned to me as a Subject Editor once more."

We checked and double checked our manuscript and references and all of them are both in the text and reference section, and no citations were used in the Results. Considering this revision, we rechecked, re-revised the formatting, and resubmitted following his recommendations of addressing the letter to him. Furthermore, specifically we added details responding his comment on the cover letter by indicating: “Although we received your editorial comments, we double checked the references and could not found any missing citation/reference, and the format is adjusted to the journal´s requirements.”. Regarding your second comment, no track changes where used ion the current MS since no changes were made.

Nevertheless, it was kind of discouraging to get rejected on the grounds of such a strange and unprecise comment; however, we acknowledged and resubmitted accordingly. Now, we are asked to mark the manuscript with the non-existent changes made, so we proceed and marked two random references to respond the unsupported comment. It is clear editors are not reading the letters nor the manuscript, we would appreciate serious consideration of our manuscript in the journal.

Please let us know if there is anything else we need to do or if the manuscript can be properly and fairly assessed and reviewed.

Best regards,

**Dr. José F. González-Maya**

Author for Correspondence

Proyecto de Conservación de Aguas y Tierras – ProCAT Colombia/Internacional.
