# Supplementary material for: Preserving the spots: Jaguar (Panthera onca) distribution and priority conservation areas in Colombia
Source: PLoS One. 2024 Mar 22;19(3):e0300375. doi: 10.1371/journal.pone.0300375 (PMC10959345; doi:10.1371/journal.pone.0300375)
Supplement: Attachment — Submitted filename: Review of MS 32228 PLOSONE .docx [file pone.0300375.s004.docx]

Review of the Manuscript Number: PONE-D-23-32228

Title: Preserving the spots of the land: Jaguar (Panthera onca) distribution and priority

conservation areas in Colombia

Authors: Machado-Aguilera et al

This paper is a comprehensive analysis of the connectivity in jaguar habitat in Colombia, a welcome addition to the urgently needed body of knowledge about this species to secure its future. I find the paper reasonably well-written, although I would recommend tighten the text overall by about 20%, and I would also suggest ensuring proper English language use. Starting with the title, the phrase “Preserving the spots of the land” does not make much sense idiomatically. The land does not have spots as such. I would recommend something like “Preserving the spots and claws: Jaguar….” Or similar

Line 52: Says: “Its large habitat and prey requirements” but I believe the authors mean something like “its requirements of extensive areas of forest and availability of large prey” or something like that, although some studies have shown that jaguars can survive in relatively small areas and feed on relatively small prey (Miranda et al 2016 J. Nat. Hist., Harmsen et al. 2011, Mamm Biol). Please resolve

Variously, authors overuse the word “the”

Line 227-228: Please expand the discussion about the category and status of the 15% protected territory

Line 252: How do you justify that a distance of merely 0.31 km is sufficient to classify an independent corridor from others? What is your benchmark for this and why?

Line 297: You say that jaguar occupied distribution has shrunk by 43%. In how much time? Since when? Any chances you can illustrate the speed of decline?

Lines 292-456: The discussion is extremely and unjustifiably long. Please reduce by 40%

Lines 457-478: Conclusions read like a selected repeat of the discussion. If no justification, please delete.

I see no need for Figure 1, which is generic and can be found elsewhere. I also believe that there may be too many maps. Can you condense into only 4 or 5?

The authors refer to habitat destruction as the main threat to jaguars. Other authors have identified direct killing and conflict with humans as the main threats, given that jaguars are known to be able to survive in suboptimal habitat if they are not hunted. I would like to see a bit of discussion regarding the synergy that these threats combine and what is the status of the other two. Preserving only the habitat may not be sufficient to secure jaguar populations, as per the empty forest paradigm and others. Please comment

Also, I believe it would be important for the authors to weigh in in the discrepancy between IUCN enlisting the species as NT, while Colombia (and many other countries) enlist the species as VU or even EN. Do the authors agree with IUCN? They only state the status of the species in IUCN and in Colombia with no other comment.
